# Characteristics of extended-spectrum β-lactamase–producing *Escherichia coli* isolated from fecal samples of piglets with diarrhea in central and southern Taiwan in 2015

**DOI:** 10.1186/s12917-017-0986-7

**Published:** 2017-03-01

**Authors:** Wan-Chen Lee, Kuang-Sheng Yeh

**Affiliations:** 1 Department of Veterinary Medicine, School of Veterinary Medicine, College of Bio-Resources and Agriculture, National Taiwan University, Taipei, 106 Taiwan; 2 National Taiwan University Veterinary Hospital, Taipei, 106 Taiwan

**Keywords:** Extended spectrum β-lactamase, *Escherichia coli*, Multilocus sequence typing

## Abstract

**Background:**

The production of extended-spectrum β-lactamases (ESBLs) confer resistance to the commonly used beta-lactam antimicrobials and ESBL–producing bacteria render treatment difficulty in human and veterinary medicine. ESBL–producing bacteria have emerged in livestock in recent years, which may raise concerns regarding possible transfer of such bacteria through the food chain. The swine industry is important in Taiwan, but investigations regarding the status of ESBL in swine are limited.

**Results:**

We collected 275 fecal swab samples from piglets with diarrhea in 16 swine farms located in central and southern Taiwan from January to December 2015 and screened them for ESBL–producing *Escherichia coli*. ESBL producers were confirmed phenotypically by combination disc test and genotypically by polymerase chain reaction and DNA sequencing. The occurrence rate of ESBL–producing *E. coli* was 19.7% (54 of 275), and all were obtained in swine farms located in southern Taiwan. *bla*_CTX-M-1-group_ and *bla*_CTX-M-9-group_ were the two *bla*_CTX-M_ groups found. *bla*_CTX-M-55_ (34 of 54; 63.0%) and *bla*_CTX-M-15_ (16 of 54; 29.6%), which belong to the *bla*_CTX-M-1-group_, were the two major *bla* gene types, whereas *bla*_CTX-M-65_ was the only type found in the *bla*_CTX-M-9 group_. Twenty-seven strains contained *bla*_TEM-1_, and the other 27 strains contained *bla*_TEM-116_. One strain found in Pingtung harbored three *bla* genes: *bla*_TEM-116_, *bla*_CTX-M-55_, and *bla*_CTX-M-65_. ESBL–producing *E. coli* exhibited a multidrug-resistant phenotype, and multilocus sequence typing revealed that the ST10 clonal complexes, including ST10, 167, 44, and 617 accounted for 35% (19 of 54) of these strains.

**Conclusions:**

ESBL-producing *E. coli* from piglets with diarrhea were isolated from swine farms located in southern Taiwan. The most commonly detected *bla* were *bla*_CTX-M-15_ and *bla*_CTX-M-55_. The ST10 clonal complexes comprised most of our ESBL-producing *E. coli* strains. Fecal shedding from swine may contaminate the environment, resulting in public health concerns; thus, continued surveillance of ESBL is essential in swine and in other food animals.

## Background

Diarrhea is a common clinical syndrome in the swine industry and may be classified into three entities. Sucking piglets usually exhibit neonatal diarrhea a few days after birth, and young piglet diarrhea occurs from the first week after birth to weaning [1]. Older piglets, commonly 2 weeks after weaning, may contract post-weaning diarrhea. Neonatal and post-weaning diarrhea are caused by pathogenic *Escherichia coli*, and causative agents of young piglet diarrhea may include transmissible gastroenteritis virus, rotavirus, coccidia, and *E. coli* [1]. Occurrence of diarrheal disease can be reduced by the vaccination of sows to let piglets obtain maternal antibodies. However, measures such as the use of antibiotic supplements in feed are also frequently practiced along with vaccination to reduce the incidence of diarrhea. If prudent usage of antibiotics is not taken into consideration, the massive, indiscriminate, and long-term use of antibiotics in veterinary practice may contribute to the selection and spread of drug-resistant bacteria [2, 3].

Production of extended-spectrum β-lactamases (ESBLs) confers resistance to the frequently used beta-lactam antimicrobial agents, including the third-generation cephalosporins such as ceftriaxone, ceftazidime, and ceftiofur. However, ESBLs are inhibited by the β-lactamases inhibitors clavulanic acid, sulbactam, and tazobactam [4]. TEM, SHV, and CTX-M-types are the three major families of ESBL [4]. All CTX-M-types enzymes are ESBLs, whereas the TEM- and SHV- types of ESBL arise by point mutation at specific residues from the natural TEM-1/TEM-2 and SHV-1 β-lactamase [5]. The production of ESBLs is mainly plasmid mediated, and such plasmids often carry genes that encode resistance to other classes of antimicrobials, such as fluoroquinolones and aminoglycosides [6]. ESBLs are widely distributed in *Enterobacteriaceae*, particularly in *E. coli*, and the rapid emergence and spread of ESBL-producing *E. coli* have been reported in food animals globally [7]. Such findings raise concerns about the possible transfer of ESBL producers through the food chain, thus presenting a hazard to public health [8].

The status of ESBL-producing *E. coli* in food animals in Taiwan has only been reported in cows [9]. Although a foot and mouth disease outbreak in 1997 had a great impact on the swine industry [10], swine are still among the most important agricultural products in Taiwan. The objective of this study is to analyze the fecal carriage of ESBL-producing *E. coli* isolated from piglets with diarrhea in 16 pig farms located in central and southern Taiwan. It is important to screen for the ESBL producers from food animals such as swine from a public health perspective.

## Methods

### Sample collection

A total of 275 fecal swab samples were collected from the piglets with diarrhea before weaning from 16 swine farms in Taiwan (one in Taichung, one in Nantou, one in Chunghua, two in Yunlin, four in Chiayi, one in Tainan, and six in Pingtung) from January to December 2015. These 16 farms belong to the same swine industry corporation. Isolating *E. coli* from feces in piglets with diarrhea and preparing “tailored vaccine” has been routinely practiced in these farms. These *E. coli* were cultured, inactivated and used as a vaccine component to feed pregnant sows. Neonatal piglets will presumably obtain maternal antibodies when sucking colostrum. Occurrence of neonatal diarrhea due to *E. coli* infection may be reduced as long as piglets have enough maternal antibody. We shared these fecal swab samples and inoculated on CHROMagar ESBL (CHROMagar, Paris, France) to screen for ESBL-producing *E. coli*. Any pink colony that appeared on the agar after incubation at 37 °C for 16–18 h was initially designated as ESBL-producing *E. coli* since they were resistant to cefotaxime and/or ceftazidime, and its identity as *E. coli* was confirmed with the RapID^TM^ ONE System (RapID^TM^, Lenexa, KS, USA). Confirmed *E. coli* strains were stored at −80 °C for further study.

### ESBL testing

*E. coli* isolates were tested phenotypically for ESBL production by combination disc tests with cefotaxime and ceftazidime (30 μg), with and without clavulanic acid (10 μg), as stated by the guidelines of the Clinical and Laboratory Standards Institute [11]. The tested *E. coli* strains were plated on Muller-Hinton agar at a concentration of 0.5 McFarland standards and grown at 35 °C for 16–18 h. A difference of 5 mm or more in the inhibition zones for at least one cefotaxime or ceftazidime/clavulanic acid combination versus the corresponding cefotaxime or ceftazidime alone was used to define an ESBL producer. *Klebsiella pneumoniae* ATCC 700603 and *E. coli* ATCC 25922 were used as the positive and negative controls, respectively [11].

### Detection of *bla* genes

The *E. coli* strains that were phenotypically confirmed to be ESBL producers were examined with polymerase chain reaction (PCR) to detect their *bla* genes. The tested strains were cultured for 16–18 h at 37 °C on tryptic soy agar plates (Difco/Becton Dickinson, Franklin Lakes, NJ, USA), and a loopful of bacterial cells was resuspended in 200 μL ddH_2_O and boiled for 10 min [12]. After centrifugation at 12,000 *g* for 10 min, the supernatant was saved as the source of template DNA for PCR. The primer sequences used to amplify *bla*_TEM_, *bla*_SHV_, *bla*_CTX-M-1-group_, *bla*_CTX-M-2-group_, *bla*_CTX-M-8-group_, *bla*_CTX-M-9-group_, and *bla*_CTX-M-25-group_, the annealing temperature, and the expected PCR product sizes are specified in Table 1. The PCR cycling program was set as follows using a LifeEco thermocycler (Bioer Technology, Hangzhou, China): initial denaturation at 95 °C for 5 min, followed by 35 cycles at 95 °C for 30 s, then the annealing temperature specified in Table 1 for 40 s, and 72 °C extension for 1 min. The reaction was then maintained at 72 °C for 10 min. Ten microliters of each PCR sample was loaded onto a 1.2% agarose gel and electrophoresed at 100 volts for 40 min. The gels were then stained with a fluorescent nucleic acid dye (Biotium, Hayward, CA, USA) and examined under a blue light LED illuminator (Smobio, Hsinchu City, Taiwan). The PCR products were then sliced from the agarose gel and subjected to further purification and sequenced by ABI 3130 x1 Genetic Analyzer (Applied Biosystems, Foster, CA, USA) in Center for Genomic Medicine, National Cheng Kung University, Tainan, Taiwan. The results were analyzed with MEGA 6.0 and examined with the NCBI BLAST program (http://www.ncbi.nlm.nih.gov/blast/) and β-lactamase database (http://www.ncbi.nlm.nih.gov/pathogens/submit-beta-lactamase).

**Table 1:** Sequences of primers used for ESBL gene detection

PCR target	primer	Sequences (5’–3’)	Annealing Tm (°C)	Predicted PCR size (bp)	Reference
*bla*_TEM_	TEM-F	TCGGGGAAATGTGCGCG	55	972	[37]
	TEM-R	TGCTTAATCAGTGAGGCACC			
*bla*_SHV_	SHV-F	GCCTTTATCGGCCCTCACTCAA	54	819	[38]
	SHV-R	TCCCGCAGATAAATCACCACAATG			
*bla*_CTX-M-1-group_	CTX-M-1-F	CCCATGGTTAAAAAATCACTGC	54	942	[39]
	CTX-M-1-R	CAGCGCTTTTGCCGTCTAAG			
*bla*_CTX-M-2-group_	CTX-M-2-F	CGACGCTACCCCTGCTATT	52	552	[40]
	CTX-M-2-R	CCAGCGTCAGATTTTTCAGG			
*bla*_CTX-M-8-group_	CTX-M-8-F	CAAAGAGAGTGCAACGGATG	52	205	[40]
	CTX-M-8-R	ATTGGAAAGCGTTCATCACC			
*bla*_CTX-M-9-group_	CTX-M-9-F	ATGGTGACAAAGAGAGTGCAAC	55	876	[26]
	CTX-M-9-R	TTACAGCCCTTCGGCGATGATT			
*bla*_CTX-M-25-group_	CTX-M-25-F	GCACGATGACATTCGGG	52	327	[40]
	CTX-M-25-R	AACCCACGATGTGGGTAGC			

### Antimicrobial susceptibility testing

The ESBL-producing *E. coli* strains were tested for susceptibility to antimicrobial agents using the disc agar diffusion method [11]. The antimicrobial agents tested included amikacin 30 μg, ampicillin 10 μg, amoxyclav 30 μg, ceftiofur 30 μg, cephalothin 30 μg, ciprofloxacin 10 μg, doxycycline 30 μg, enrofloxacin 5 μg, florfenicol 30 μg, gentamicin 30 μg, nalidixic acid 30 μg, streptomycin 10 μg, co-trimoxazole 25 μg, tetracycline 10 μg; all of the discs were purchased from Oxoid (Oxoid, Hampshire, UK).

### *E. coli* genotyping

Our *E. coli* strains were analyzed genotypically by multilocus sequence typing. DNA fragments derived from *adk*, *fumC*, *gyrB*, *icd*, *mdh*, *purA*, and *recA* were amplified by PCR, sequenced, and then uploaded to the MLST website (http://enterobase.warwick.ac.uk/) for comparison [13]. Phylogenetic analysis was performed using BioNumerics Software version 7.0 (Applied Maths, Sint-Martens-Latem, Belgium).

## Results

**Fig. 1:**
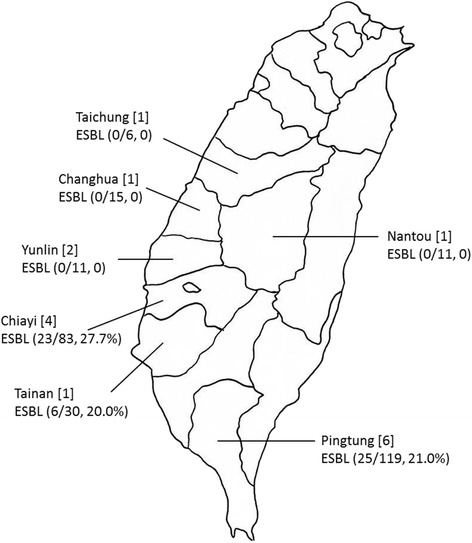
Geographic distribution of the swine farms in Taiwan included in this study. The number of square brackets indicates the number of swine farms included from each region. The number and occurrence rate of ESBL-producing *E. coli* are denoted in parentheses

Fifty-four samples exhibited pink colonies on CHROMagar ESBL, initially indicating an identity of ESBL–producing *E. coli*. All of these strains were then confirmed biochemically with RapID^TM^ ONE System as *E. coli*, and they were not hemolytic when grown on blood agar. These 54 strains exhibited the ESBL phenotype when assayed by combination disc tests. From our results, we did not detect any ESBL–producing *E. coli* in diseased piglets from any of the five swine farms in Taichung, Nantou, Chunghua, and Yunlin, which were located in central Taiwan. ESBL–producing *E. coli* were all obtained in swine farms in southern Taiwan, including Chiayi, Tainan, and Pingtung, with the exception of one farm in Chiayi (farm ID CY-3) and one in Pingtung (farm ID PT-3). Geographic distribution of the swine farms and occurrence of ESBL were indicated in Fig. 1. Overall, the occurrence rate of ESBL-producing *E. coli* was 19.7% (54 of 275). Table 2 lists the occurrence of ESBL-producing *E. coli* in 16 swine farms.

**Table 2:** Occurrence of ESBL–producing *E. coli* in 16 farms

Farm location	Farm ID	No. of fecal samples	No. of ESBL-producing *E. coli*	Occurrence (%)
Taichung	TC-1	6	0	0
Changhua	CH-1	15	0	0
Nantou	NT-1	11	0	0
Yunlin	YL-1	5	0	0
	YL-2	6	0	0
Chiayi	CY-1	40	19	47.5
	CY-2	8	2	25.0
	CY-3	29	0	0
	CY-4	6	2	33.3
Tainan	TN-1	30	6	20.0
Pingtung	PT-1	10	2	20.0
	PT-2	36	16	44.4
	PT-3	37	0	0
	PT-4	17	2	11.8
	PT-5	11	3	27.3
	PT-6	8	2	25.0

Table 3 lists the *bla* genes and sequence type of ESBL-producing *E. coli. bla*_CTX-M-1-group_ and *bla*_CTX-M-9-group_ were the two *bla*_CTX-M_ groups found in ESBL-producing *E. coli*. The *bla*_CTX-M-1-group_ contained *bla*_CTX-M-55_ (34 of 54, 63.0%) and *bla*_CTX-M-15_ (16 of 54, 29.6%), whereas *bla*_CTX-M-65_ was the only type found from the *bla*_CTX-M-9 group_. All 54 strains contained *bla*_TEM_; 27 strains had *bla*_TEM-1_ and the other 27 strains contained *bla*_TEM-116_. One strain found in Pingtung harbored *bla*_TEM-116_, *bla*_CTX-M-55_, and *bla*_CTX-M-65_. The *bla*_CTX-M-2-group_, *bla*_CTX-M-8-group_, *bla*_CTX-M-25-group_, and *bla*_SHV_ types of ESBL were not detected in this study.

**Table 3:** The *bla* genes and sequence type of ESBL-producing *E. coli*

Farm location	*bla* gene	Sequence Type
Chiayi	*bla*_TEM-1_ + *bla*_CTX-M-15_ (*n* = 9)	4981 (*n* = 5), 1638 (*n* = 1), 3268 (*n* = 1), NT^a^ (*n* = 2)
	*bla*_TEM-116_ + *bla*_CTX-M-15_ (*n* = 3)	1638 (*n* = 1), NT (*n* = 2)
	*bla*_TEM-1_ + *bla*_CTX-M-55_ (*n* = 5)	167 (*n* = 4), NT (*n* = 1)
	*bla*_TEM-6_ + *bla*_CTX-M-55_ (*n* = 6)	4981 (*n* = 1), 349 (*n* = 2), 44 (*n* = 2), NT (*n* = 1)
Tainan	*bla*_TEM-1_ + *bla*_CTX-M-55_ (*n* = 2)	NT (*n* = 2)
	*bla*_TEM-116_ + *bla*_CTX-M-55_ (*n* = 3)	457 (*n* = 2), 617 (*n* = 1)
	*bla*_TEM-116_ (*n* = 1)	NT (*n* = 1)
Pingtung	*bla*_TEM-1_ + *bla*_CTX-M-15_ (*n* = 2)	617 (*n* = 2)
	*bla*_TEM-1_ + *bla*_CTX-M-55_ (*n* = 9)	10 (*n* = 3), 648 (*n* = 1), 69 (*n* = 3), 617 (*n* = 1), NT (*n* = 1)
	*bla*_TEM-116_ + *bla*_CTX-M-15_ (*n* = 2)	10 (*n* = 1), 349 (*n* = 1)
	*bla*_TEM-116_ + *bla*_CTX-M-55_ (*n* = 8)	NT (*n* = 1), 10 (*n* = 1), 457 (*n* = 2), 617 (*n* = 1), 167 (*n* = 1), 69 (*n* = 1), 38 (*n* = 1)
	*bla*_TEM-116_ + *bla*_CTX-M-55_ + *bla*_CTX-M-65_ (*n* = 1)	NT (*n* = 1)
	*bla*_TEM-116_ (*n* = 3)	44 (*n* = 1), 167 (*n* = 1), NT (*n* = 1)

^a^: *NT* new type, there was no comparison standard in the databank

The results of the antibiotic susceptibility testing of the ESBL-producing *E. coli* isolated from Chiayi, Tainan, and Pingtung are shown in Table 4. The susceptibility testing showed that all 54 ESBL positive isolates were resistant to five antibiotics: ampicillin, cephalothin, ceftiofur, tetracycline, and enrofloxacin. Amikacin and gentamicin were active against 31 strains (57.4%) and 17 strains (31.5%) of ESBL producers, respectively. Overall, ESBL-producing *E. coli* exhibited a multi-drug-resistant phenotype.

**Table 4:** Antimicrobial susceptibility test of ESBL–producing *E. coli*

	Chiayi, *n* = 23 (%)			Tainan, *n* = 6 (%)			Pingtung, *n* = 25 (%)			Total, *N* = 54 (%)		
Antibiotic discs used	S^a^	I^b^	R^c^	S	I	R	S	I	R	S	I	R
Ampicillin	0	0	23 (100.0)	0	0	6 (100.0)	0	0	25 (100.0)	0	0	54 (100.0)
Amoxicillin/clavulanic acid	3 (13.0)	8 (34.8)	12 (52.2)	2 (33.3)	1 (16.7)	3 (50.0)	6 (24.0)	11 (44.0)	8 (32.0)	11 (20.4)	20 (37.0)	23 (42.6)
Cephalothin	0	0	23 (100.0)	0	0	6 (100.0)	0	0	25 (100.0)	0	0	54 (100.0)
Ceftiofur	0	0	23 (100.0)	0	0	6 (100.0)	0	0	25 (100.0)	0	0	54 (100.0)
Amikacin	15 (65.2)	8 (34.8)	0	5 (83.3)	1 (16.7)	0	11 (44.0)	3 (12.0)	11 (44.0)	31 (57.4)	12 (22.2)	11 (20.4)
Gentamicin	12 (52.2)	3 (13.0)	8 (34.8)	1 (16.7)	0	5 (83.3)	4 (16.0)	0	21 (84.0)	17 (31.5)	3 (5.6)	34 (62.9)
Streptomycin	0	1 (4.4)	22 (95.6)	0	0	6 (100.0)	0	1 (4.0)	24 (96.0)	0	2 (3.7)	52 (96.3)
Doxycycline	3 (13.0)	10 (43.5)	10 (43.5)	2 (33.3)	2 (33.3)	2 (33.3)	2 (8.0)	9 (36.0)	14 (56.0)	7 (13.0)	21 (38.9)	26 (48.1)
Tetracycline	0	0	23 (100.0)	0	0	6 (100.0)	0	0	25 (100.0)	0	0	54 (100.0)
Nalidixic acid	1 (4.4)	0	22 (95.6)	0	0	6 (100.0)	2 (8.0)	0	23 (92.0)	3 (5.6)	0	51 (94.4)
Ciprofloxacin	1 (4.4)	0	22 (95.6)	0	0	6 (100.0)	3 (12.0)	0	22 (88.0)	4 (7.4)	0	50 (92.6)
Enrofloxacin	0	0	23 (100.0)	0	0	6 (100.0)	0	0	25 (100.0)	0	0	54 (100.0)
Florfenicol	0	1 (4.4)	22 (95.6)	0	0	6 (100.0)	0	4 (16.0)	21 (84.0)	0	5 (9.3)	49 (90.7)
Co-trimoxazole	6 (26.1)	0	17 (73.9)	0	0	6 (100.0)	2 (8.0)	0	23 (92.0)	8 (14.8)	0	46 (85.2)

^a^: susceptible; ^b^: intermediate resistant; ^c^: resistant

**Fig. 2:**
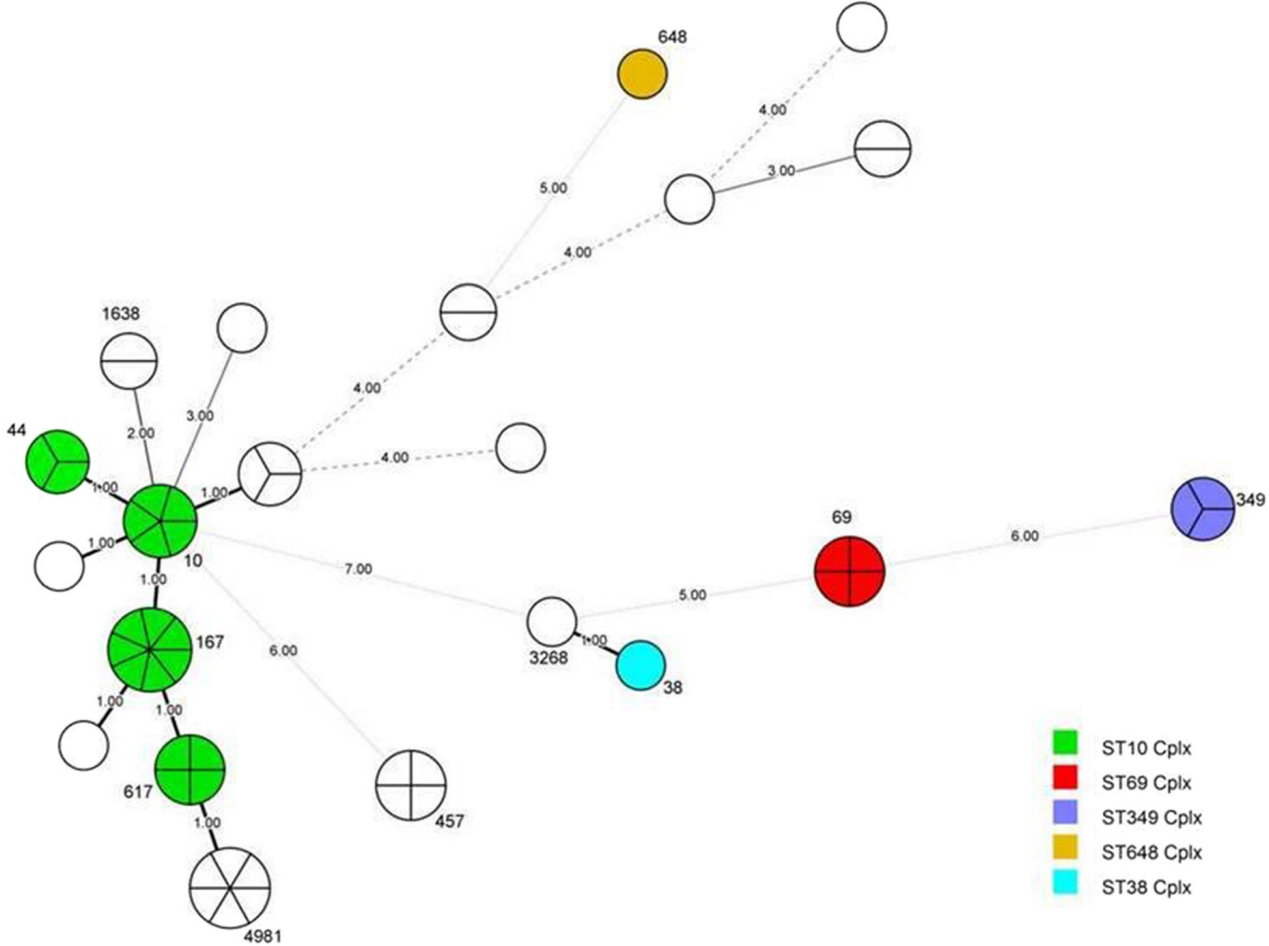
Minimal spanning tree (MSTree) of ESBL-producing *E. coli*. Each circle indicates one ST, subdivided into one sector for each isolate, and bordered by the ST number. White circles or sectors without an ST number denote a lack of comparison standard in the current databank. The numbers on the connecting line between STs within the MSTree indicate the number of different alleles. Solid lines represent an allele difference of 3 or less, whereas dotted lines and faint lines indicate an allele difference of 4 or more

The most frequently seen sequence type of ESBL-producing *E. coli* was ST167 (ST10 clonal complex; 7 of 54; 13.0%), followed by ST4981 (6 of 54; 11.1%) and ST10 (ST10 clonal complex; 5 of 54; 9.3%). There were four strains of ST617 (ST10 clonal complex, 4/54, 7.4%), ST457, and ST69 (ST69 clonal complex) and three strains of ST44 (ST10 clonal complex; 3 of 54; 5.6%) and ST349 (ST349 clonal complex). ST1638 had two strains (2 of 54; 3.7%). ST38 (ST38 clonal complex), ST3268, and ST648 (ST648 clonal complex) had only one strain each. Nonetheless, we still had 13 strains whose sequence types were not matched to any type in the current databank. Figure 2 indicates the minimal spanning tree of the ESBL-producing *E. coli* STs based on the degree of allele sharing.

## Discussion

The ESBL-producing *E. coli* were all obtained from the swine farms located in southern Taiwan. There were five swine farms in central Taiwan (Taichung, Changhua, Yunlin, and Nantou) that participated in our study, and only 43 fecal samples (43 of 275; 15.6%) were collected from piglets with diarrhea and screened for ESBL. Although the scale of these farms was similar to that of those located in southern Taiwan, the hygienic procedures or disease control management of individual farms may contribute to such differences in diarrheal cases. The specificity of this chromogenic agar was 100% because all of the pink colonies, indicative of ESBL-producing *E. coli*, were phenotypically and genotypically positive for ESBL. A previous report also suggested the high sensitivity and specificity of CHROMagar ESBL in the detection of clinical ESBL-producing *Enterobacteriaceae* [14]. However, our strategy may also lose some ESBL producers that could grow on blood agar or MacConkey agar but not on CHROMagar ESBL.

The occurrence of ESBL-producing *E. coli* in food animals has been increasing around the world [2]. For example, more than 40% of the ESBL-producing *E. coli* were detected from piglets with post-weaning diarrhea in Heilongjiang Province, China [15]. The authors also compared their findings with those of a similar study in healthy pigs in China and concluded that ESBL-producing *E. coli* were more commonly found in sick animals [16]. Because we did not investigate the prevalence of ESBL in a healthy swine population, there was no basis of comparison for healthy and diseased swine in Taiwan. Although diseased pigs are not likely to enter slaughter or market, fecal shedding from such pigs can contaminate the piggery environment and provide a reservoir for the exchange of drug-resistance genes [17].

TEM-116–producing *E. coli* was first identified in Korean hospitals in a nationwide survey in 2002 [18]. Consequently, a high prevalence of TEM-116 was also reported in Spain [19]. In animals, TEM-116–producing *E. coli* has been detected in dogs [20, 21]. Our results, to the best of our knowledge, demonstrate for the first time the presence of *bla*_TEM-116_ genes in the ESBL–producing *E. coli* from porcine origin. Although most of our TEM-116–containing strains also had CTX-M, we did find four *E. coli* strains that harbored only TEM-116 that exhibited an ESBL phenotype. ESBL producers within the CTX-M group are becoming more common [22, 23]. In Europe, CTX-M-1 is broadly disseminated in animals, whereas CTX-M-14 is most prevalent in animals in Asian countries [8]. The most frequently found CTX-M types in our study were CTX-M-15 and CTX-M-55, whereas CTX-M-14 was reported in healthy and diseased swine in Korea and China [15, 16, 24]. CTX-M-55 was first isolated from patients in a hospital in Thailand; this novel CTX-M type was derived from CTX-M-15, with only a single substitution of valine instead of alanine at residue 77 [25]. The incidence of CTX-M-55 has been reported to exceed that of CTX-M-15 in outpatient infection cases in Chinese county hospitals [26]. Thus, the authors of that study hypothesized an animal-human transfer of CTX-M-55 because most of these outpatients in county hospitals live in rural areas and thus have more chances to come into contact with infected food animals and farm sewage [26]. In our study, CTX-M-55 was the predominant ESBL type isolated from swine with diarrhea in southern Taiwan. CTX-M-55–producing *E. coli* has also been detected in the milk of cows with clinical mastitis in the same region [9]. It is conceivable that ESBL-producing *E. coli* that possess CTX-M-55 have spread to the environment. One strain obtained in Pingtung possessed CTX-M-65 in addition to CTX-M-55 and TEM-116. Although the detection rate of CTX-M-65 was low compared to those of CTX-M-55 and CTX-M-15, the presence of CTX-M-65 has been reported in humans, animals, and vegetables [15, 24, 27]. These findings underscore the importance of screening and investigation of the genotypes of ESBL producers in food animals on a regular basis. Our investigation did not detect any CTX-M-8 or CTX-M-25, which were also not detected in previous studies [15, 28].

Antimicrobial susceptibility test revealed a high frequency of the resistance of ESBL-producing *E. coli* to most antimicrobial agents. Inappropriate use or overuse of antimicrobial agents, including third-generation cephalosporins, may be associated with the emergence of ESBL-producing *E. coli* in swine [29]. The selection of CTX-M-producing *E. coli* in swine by treatment with ceftiofur has been documented [30]. It is worthwhile to consider banning the use of third-generation of cephalosporins such as ceftiofur in food animals to decrease the occurrence of ESBL producers. For example, the occurrence of ESBL-producing *E. coli* was reduced when third-generation cephalosporins were banned in the Danish pig industry [31]. Forty-two percent of ESBL-producing *E. coli* were resistant to amoxicillin/clavulanic acid. Possible reasons that account for this phenotype may include hyper production of chromosomal class C β-lactamase, possession of plasmid-mediated TEM enzymes, production of oxacillinases, or production of inhibitor-resistant TEM by these isolates [32]. In addition, plasmid mediated AmpC may also cause resistance to amoxicillin/clavulanic acid [8].

The ST10 clonal complexes (ST10, 167, 44, 617) comprised most of our ESBL-producing *E. coli* strains. There were 13 strains that did not match any ST in the current database; however, six of these had only a one to three-allele difference from ST10 clonal complexes. It is fair to say that ST10 was the dominant clonal complex in our study. A recent investigation indicated that ESBL-producing *E. coli* were commonly isolated from river waters in southern Taiwan and that ST10 and ST58 was the most frequently found clonal complexes [33]. The authors of that study also observed a substantial association of these ESBL-producing *E. coli* with the presence of chicken farms at that region. Geographically, food animal farms, including swine, chicken, and cattle, are primarily situated in southern Taiwan. It is conceivable that livestock may spread ESBL-producing *E. coli* from feces, thus contaminating the environment. We did not detect ESBL-producing *E. coli* ST131 (O25:H4) that possessed CTX-M-15, a leading cause of urinary tract infections and bacteremia in human medicine globally, in our study. However, swine and other food animals may play a role as vectors in the transmission of bacteria to humans [34], so continued surveillance of food animals for ESBL-producing *E. coli* is essential.

Our study has some limitations. Fecal samples from healthy piglets were not collected and there was no comparison for the occurrence of ESBL-producing *E. coli* between the healthy and diseased populations. The virulence factors like K88, K99 or 987 P fimbriae genes in our ESBL-producing *E. coli* isolates were not screened and they were not hemolytic when grown on blood agar. It is possible that the ESBL-producing *E. coli* in the present study was not the causative agent for the diarrhea of these piglets. We did not detect if these *E. coli* isolates produced AmpC-β-lactamases, which also hydrolyze third-generation cephalosporins. AmpC-producing *E. coli* were also found in increasing numbers in food-producing animals [8]. In addition, profiles of resistant plasmids were not characterized in our study. Plasmid analysis methods like PCR-based replicon typing could assign the incompatibility (Inc) groups [35], whereas replicon sequence typing could discriminate IncF plasmid variants [36]. Inclusion of plasmid characterization could have provided insights into the epidemiology of the ESBL plasmid in our study.

## Conclusions

ESBL-producing *E. coli* from piglets with diarrhea were isolated from swine farms located in Chiayi, Tainan, and Pingtung. *bla*_CTX-M-15_ and *bla*_CTX-M-55_ were the most commonly detected *bla* genes. The ST10 clonal complexes comprised most of our ESBL-producing *E. coli* strains. Fecal shedding from swine may contaminate the environment, from a public health perspective, continued surveillance of ESBL is essential in swine and in other food animals.

## Abbreviations

ATCC: American type culture collection
BLAST: Basic local alignment search tool
CTX-M: Cefotaximase-Munich
ESBL: Extended-spectrum β-lactamase
h: hour
MEGA 6.0: Molecular evolutionary genetics analysis software version 6.0
min: minute
MLST: Multilocus sequence typing
NCBI: National center for biotechnology information
PCR: Polymerase chain reaction
s: second
SHV: Sulfhydryl variable
ST: Sequence type
TEM: Temoneira
